# Randomized Clinical Trial of a Self-care and Communication Intervention for Parents of Adolescent/Young Adults Undergoing High-Risk Cancer Treatment: A Report From the Children’s Oncology Group

**DOI:** 10.1097/NCC.0000000000001038

**Published:** 2021-11-24

**Authors:** Joan E. Haase, Kristin Stegenga, Sheri L. Robb, Mary C. Hooke, Debra S. Burns, Patrick O. Monahan, Timothy E. Stump, Amanda K. Henley, Paul R. Haut, Brooke Cherven, Lona Roll, Anne-Marie Langevin, Rita H. Pickler, Karen Albritton, DeAnna Hawkins, Erin Osterkamp, Pauline Mitby, Jackie Smith, Virginia R. Diaz, Erica Garcia-Frausto, Margo Moore

**Affiliations:** Author Affiliations: Indiana University School of Nursing (Drs Haase and Robb, and Ms Henley), Indianapolis; Children’s Mercy Hospital (Dr Stegenga), Kansas City, Missouri; Children’s Minnesota (Dr Hooke and Ms Mitby); and University of Minnesota School of Nursing (Dr Hooke), Minneapolis; Purdue School of Engineering and Technology, IUPUI (Dr Burns), Indianapolis, Indiana; Department of Biostatistics & Health Data Science Indiana University Schools of Medicine & Public Health (Dr Monahan), Indianapolis; Department of Biostatistics, Indiana University School of Medicine (Mr Stump), Indianapolis; Riley Hospital for Children at IU Health, Department of Pediatrics, Indiana University School of Medicine (Dr Haut), Indianapolis; Aflac Cancer & Blood Disorders, Children’s Healthcare of Atlanta (Dr Cherven), Georgia; Emory University School of Medicine (Dr Cherven), Atlanta, Georgia; University of Texas Health Science Center at San Antonio (Ms Roll, Dr Langevin, and Ms Diaz); The Ohio State University (Dr Pickler), Columbus; Cook Children’s Medical Center (Dr Albritton), Fort Worth, Texas; Cincinnati Children’s Hospital Medical Center, Ohio (Dr Hawkins, Ms Osterkamp, and Ms Moore); Piedmont Healthcare, Inc (Ms Smith), Atlanta, Georgia; and Jazz Pharmaceuticals (Dr Garcia-Frausto), Palo Alto, California.

**Keywords:** Adolescents, Cancer, Communication, Family caregiver, Parents, Self-management, Stress, Young adults

## Abstract

Parents of adolescents and young adults (AYAs) with cancer offer primary support to their children and often experience their own high levels of distress, affecting parent-AYA communication and quality of life.

**Objective:**

To reduce parent distress and improve communication during high-risk cancer treatment, we examined efficacy of a self-care and communication intervention for parents and indirect benefit for AYAs receiving a therapeutic music video (TMV) intervention.

**Methods:**

In this study, we conducted a multisite, randomized controlled trial with AYAs and parents enrolled as dyads (n = 110). Parents were randomized to intervention or low-dose control; all AYAs received TMV. Data collection occurred at baseline, 2 weeks post intervention (T2), and 90 days post intervention (T3).

**Results:**

There were no significant between-group differences on primary outcomes for parents or AYAs. We did find significant differences favoring the parent intervention group on parenting confidence at T2 and marginally better outcomes for family adaptability/cohesion at T3. Both groups exhibited significant within-group improvement for parent distress (state anxiety, T3; perceived stress, T2 and T3; mood, T3), state anxiety (T2) intervention only, and family strengths control group only. Qualitative data demonstrate the parent intervention raised self-awareness and parent confidence in the short term.

**Conclusion:**

Parents found their intervention helpful. Absence of significant results may be due to short intervention duration, need for tailored content, underpowered sample, and potential indirect parent benefit from AYA participation in TMV. The parent intervention did not provide an indirect benefit for AYAs.

**Implications for Nursing:**

Parents identified their own need for communication and support from nurses. Nurses can optimize AYA care by attending to parent needs through supportive listening and encouraging self-care.

According to the US Cancer Statistics for 2020, it is estimated that approximately 30 700 parents of adolescents and young adults (AYAs) (ages 15-29 years) will learn that their child has cancer, and of those, approximately 2750 will experience the death of their child due to cancer or its resulting treatment.^1^ While parents of AYAs with high-risk cancers offer primary support to their children during treatment, they often experience their own high levels of distress, including heightened anxiety, depressed mood, and stress.^2–7^ The immediate effect of parent distress includes diminished parent-AYA communication, family function (adaptability, cohesion), and quality of life.^5,8–11^

Even under the best circumstances, parent-AYA communication can be challenging. Throughout adolescence, young people experience an increase in their need for privacy and autonomy from their parents—a process referred to as individuation.^12,13^ Health individuation occurs when AYAs establish their autonomy without having to sacrifice a relationship with their parents. Cancer diagnosis and resulting treatment can interrupt and complicate the normal process of individuation as AYAs are thrust into a higher level of dependence on their parents, challenging their need for independence, while parents turn their focus toward survival and understandably may become hypervigilant in an effort to protect their AYA child.^13–16^

Open parent-AYA communication about stressful life events, including high-risk cancer treatment, is associated with better short- and long-term emotional adjustment outcomes.^17–20^ However, communication between parent and AYA about cancer-related concerns and goals for the future is often difficult, stressful, and/or avoided in an effort to diminish distress for both.^12,14,20–24^ These challenges affect the resilience and quality of life for both parent and AYA. Importantly, all of the extant literature cited here included adolescents in their sample; however, the upper age range for most articles was 18 to 21 years indicating an absence of information for young adults.

Haase et al’s^25^ Resilience in Illness Model (RIM), the guiding framework for research conducted through the Children’s Oncology Group (COG) Nursing Discipline,^26^ includes 2 risk and 5 protective factors that either inhibit or promote resilience resolution, self-transcendence, and quality of life (ie, sense of well-being; Figure 1). Strong “family environment,” which includes family cohesion, adaptability, communication, perceived family strengths, and social support, is a protective factor against behavioral and psychological problems in children/adolescents developing during high-risk cancer treatment.^9,10,17,19,20,27^ To promote these protective factors for the family, it is important that parents receive interventions to help them manage their own emotional distress and learn strategies for supportive communication with their AYAs. To address this need and on the basis of findings from our previous efficacy trial of a therapeutic music video (TMV) intervention for AYAs undergoing hematopoietic stem cell transplant for cancer (COG ANUR0631),^28^ we developed a nurse delivered “self-care and communication intervention” for parents of AYAs undergoing high-risk cancer treatment and received funds to examine its efficacy through a National Institutes of Health competing continuation trial (COG ANUR1131). The parent intervention was delivered along with the existing TMV intervention for AYAs. All AYAs in the trial received the TMV intervention. Parents were randomized to either the intervention or an attention control condition.

**Figure 1:**
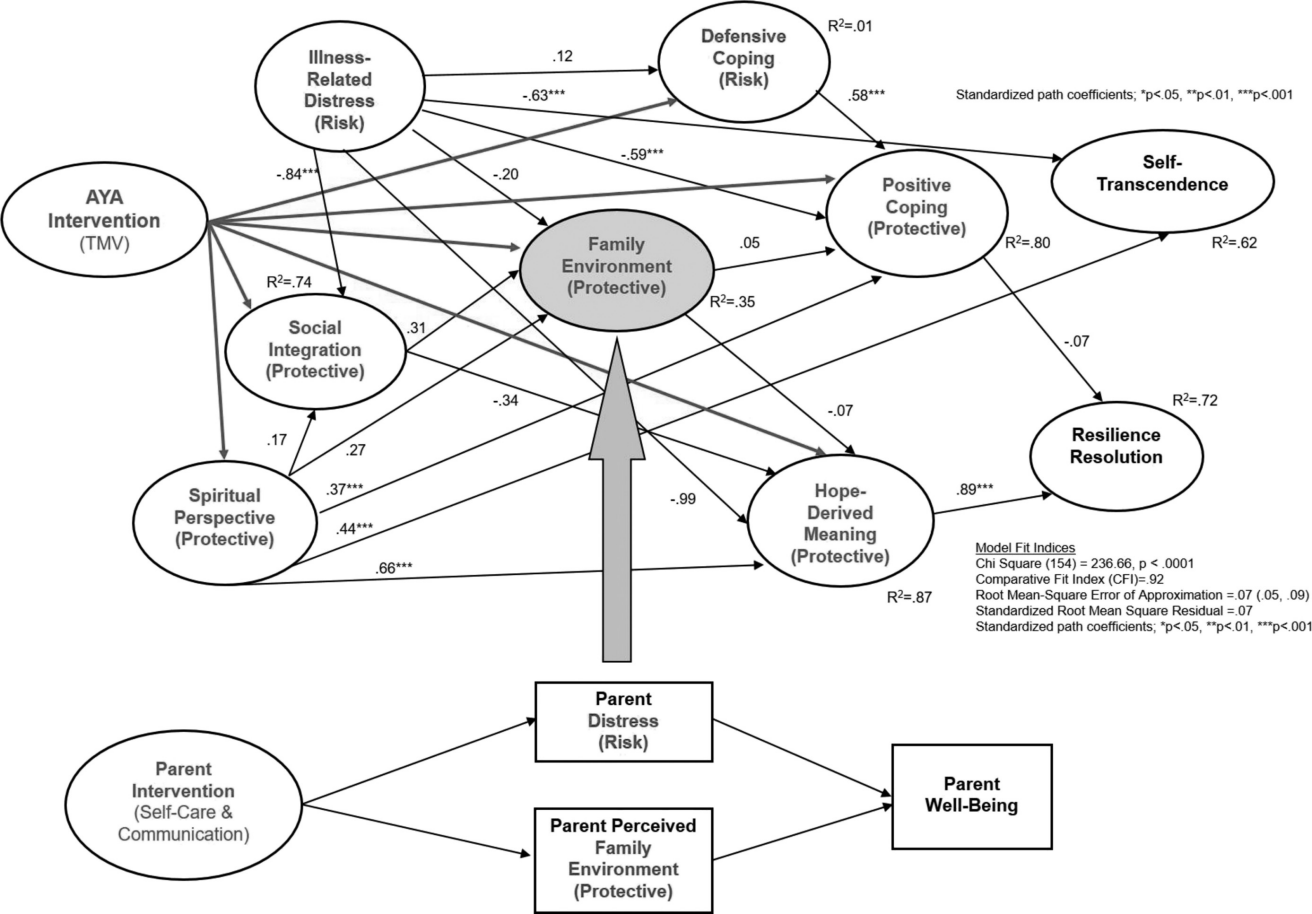
Conceptual framework: interaction of AYA and parent interventions on Resilience in Illness Model protective factors. AYA, adolescent and young adult; TMV, therapeutic music video.

The TMV is a music therapist–delivered intervention that uses songwriting and video production to help AYAs explore, identify, and express what is important to them during treatment for a life-threatening illness. In our previous trial, AYAs who received the TMV reported significantly better positive coping post intervention, and significantly better social integration and family environment 100 days post transplant.^28^ In addition, some parents derived an indirect benefit from the AYA-directed TMV intervention, gaining a deeper understanding of their AYAs’ perspectives about their cancer treatment.^29^ However, these earlier findings also indicated parents want and need strategies to help them manage their own distress, respect their AYAs’ autonomy, and open conversations about what is important and meaningful to their AYAs during cancer treatment.

On the basis of Haase et al’s^25^ RIM, which served as the measurement model and guiding theoretical framework for this trial, we hypothesized that adding a parent intervention (delivered in tandem with the TMV) would lower parent distress and improve parent perceptions about their “family environment” (Figure 1). Content for the parent intervention was rooted in the RIM protective factor, “family environment,”^25^ and was also based on findings from qualitative interviews with parents in our initial trial,^29^ as well as consultations with our parent advisor, and further substantiated by descriptive research documenting the experiences and needs of parents of AYAs with cancer.^30–33^

In this trial, we hypothesized that parents of AYAs who received the parent-communication intervention would experience less distress (anxiety, mood, perceived stress), improved “family environment,” and greater well-being 2 weeks post intervention (T2) and 90 days post intervention (T3). As a secondary outcome, we hypothesized that parents who received the intervention would have greater parenting confidence at T2 and T3. As shown in Figure 1, AYA participant outcomes were measured in both the intervention and control groups to determine whether the parent-focused communication intervention would strengthen AYA perceptions of “family environment” and result in an additional indirect benefit for AYAs beyond that seen with TMV alone in our previous trial. For AYAs, we hypothesized that AYAs whose parents received the communication intervention would experience greater “family environment,” and greater “positive coping,” “hope-derived meaning,” and “resilience resolution.”

## Methods

### Participants

We recruited participants from 7 COG institutions (2011-2016). After obtaining scientific and institutional review board approvals at each site, oncology staff introduced the study to potentially eligible parents and AYAs. Study personnel then provided interested parents and AYAs with detailed information about the study before obtaining informed consent/assent. Parents and AYAs were recruited as dyads, requiring consent/assent from both.

The age range for AYAs in this trial was 11 to 24 years. At the time of our first TMV trial (2005-2011), AYA oncology was only beginning to emerge as a subspecialty and a specific age range was not established until 2006.^34^ Because this trial was a competing continuation of a previously funded trial, it was important to maintain the original age criteria. However, the age range for the AYA cancer population has evolved since then, with the most recent definition in the United States specifying 15 to 39 years old.^35^

For this trial, eligibility criteria included (1) AYAs who were aged 11 to 24 years inclusive at the time of consent; (2) AYAs who had an initial or relapsed cancer diagnosis; (3) AYAs who met at least 1 of 3 criteria indicating potentially high palliative care or end-of-life needs, namely, any high-risk cancer (ie, metastatic or stage IV), receiving moderate- to high-intensity chemotherapy during 3 to 5 consecutive days in an inpatient or outpatient setting, or a diagnosis with an estimated event-free survival of 50% or less; (4) AYAs who were able to participate in sessions as indicated by a Karnofsky/Lansky score of 50 or greater; (5) 1 consistent parent who was willing and available to participate in all parent and evaluation sessions; (6) AYAs who were not married and had no children; and (7) both AYAs and parents who were able to read, understand, and speak English. Exclusion criteria included cancer diagnosis not common to AYAs and/or physician determination of cognitive impairments precluding ability to complete measures. Adolescent and young adult participants received gift cards after completing T2 and T3 measures ($20 and $30, respectively). Parent participants were not compensated for their participation in the trial.

### Procedures

**Figure 2:**
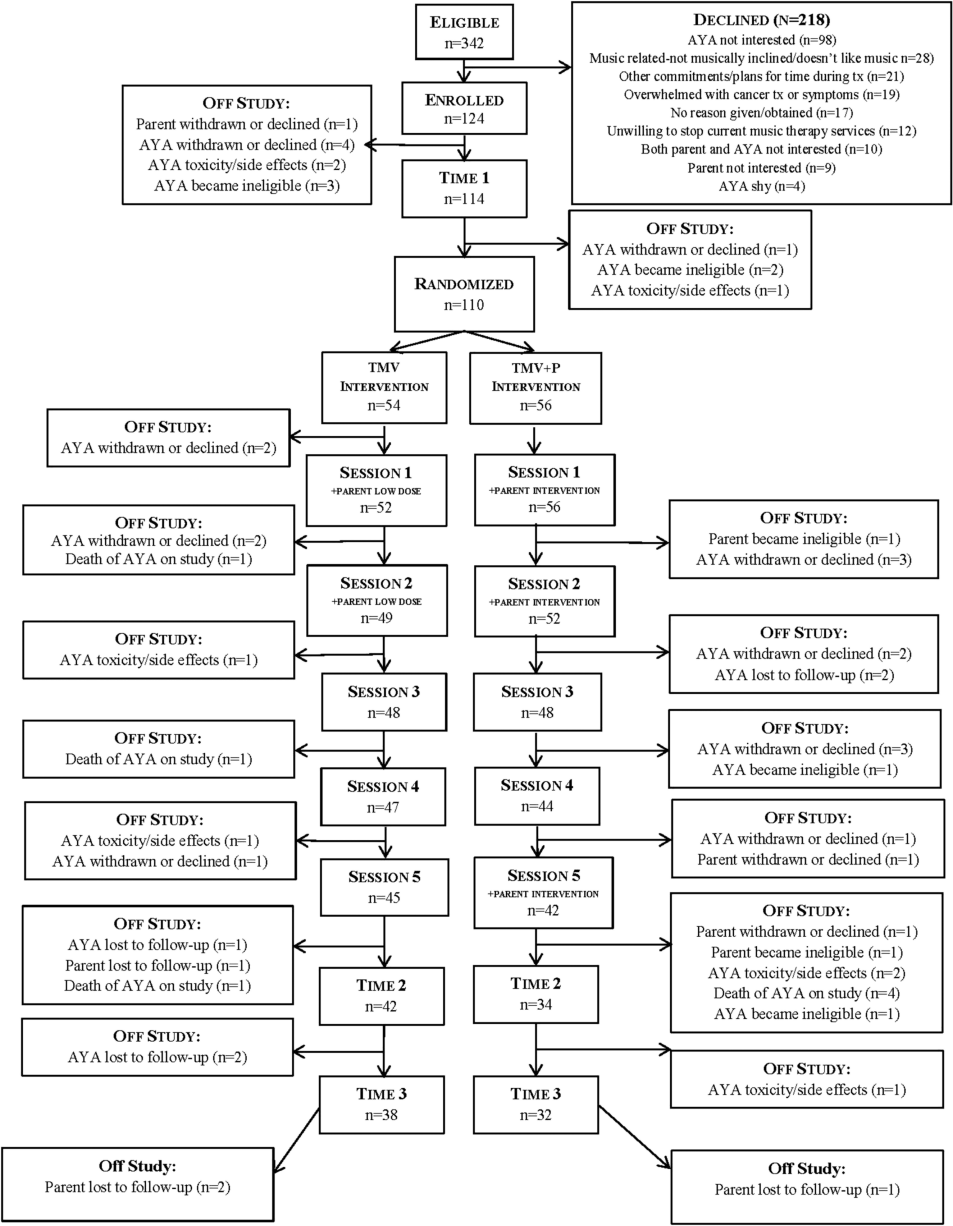
CONSORT diagram. AYA, adolescent and young adult; TMV, therapeutic music video.

Participants completed study measures at 3 time points using laptop computers connected to a secure Web-based server. A trained evaluator, masked to randomization status, provided instruction on laptop use and remained available to answer questions during each evaluation session. Participants completed baseline measures (T1) after informed consent and before session 1. Participants were then immediately randomized to the parent intervention or attention control condition using stratified blocks (by site and 3 age groups: 11-13, 14-17, and 18-24 years). Parent participants received three 60-minute parent-only sessions scheduled to correspond with AYA sessions 1, 2, and 5. Parent participants in the attention control group received 2 sessions that corresponded with AYA sessions 1 and 2 (Figure 2). Parents completed time 2 (T2) measures 2 weeks after parent session 3 and time 3 (T3) measures 90 days post intervention. All AYA participants received five 60-minute TMV intervention sessions within a 6- to 8-week period. Adolescents and young adults completed T2 measures after AYA session 5 and T3 measures 90 days post intervention. Study team members trained in qualitative techniques conducted audio-recorded interviews with parents who received the intervention after T3 measures. Intervention and evaluation fidelity strategies included standardized training, manualized protocols, audio-recorded sessions for quality assurance (QA) monitoring, computerized QA checklists, and bimonthly intervention and evaluation team conference calls.^36–38^

### Study Conditions

Parents were randomized to the intervention or attention control group. All AYAs received the same TMV intervention regardless of parent randomization. Timing of parent sessions corresponded with AYA sessions (Figure 2), with parent sessions delivered in a private space. Similarly, TMV sessions were delivered in a private space and were independent of parent intervention sessions. All sessions occurred during inpatient hospitalization or outpatient clinic visits, depending on timing and requirements of the specific chemotherapy protocol the AYA was receiving (exception was parent control session 2, delivered by telephone). Self-monitoring and external QA monitoring were performed to ensure that interventionists consistently followed protocol for assigned study conditions.

#### PARENT SELF-CARE AND COMMUNICATION INTERVENTION

Parents assigned to the intervention group received 3 tailored 60-minute sessions with a trained nurse intervener who was an advanced practice nurse or a registered nurse with a minimum of 3 years of experience in pediatric/adolescent oncology. Intervention content was structured based on Haase et al’s^25^ RIM and Robb’s^39,40^ Contextual Support Models: (1) “Managing the Chaos: Self Care as the First Step to Caring for Your AYA,” (2) “Relationship Support: How to Listen to and Encourage Your AYA to Talk,” and (3) “Strategies for AYA Autonomy Support: Understanding AYA’s Ways of Coping.” All sessions included check-in and goals, content tailored to parent-expressed information desires/needs, skills practice/role play, reflection on and reinforcement of learning, and take-home tip sheets and prescribed skills practice plans (Table 1).

**Table 1:** Parent Self-care and Communication Intervention

Session 1		
Structure	Content	Printed Material Content
Introduction	- Discuss program goals and structure.	*Tip Sheet 1.1: Relaxation Exercises* Deep breathing, progressive muscle relaxation, imagery Prayer (listed but not practiced) *Tip Sheet 1.2: Additional Strategies* Asking for help, sharing your experience, scheduling fun/laughter, staying connected, listening to music, journaling *Relaxation Prescription Form* Prescription for use and tracking form
Reflecting on AYAs’ experience	- Conversations centered on AYAs’ response to cancer diagnosis/treatment, beliefs, and values.	
Managing distress	- Discuss managing own stress as a way to care for AYA. - Review *Tip Sheet 1: Relaxation Exercises*. - Practice 1 selected strategy. - Identify/discuss barriers for home use.	
Wrap-up	- Review *Tip Sheet 2: Additional Ways to Care for Yourself*. - Complete *Relaxation Prescription Form*. - Identify relaxation strategy for home use. - Specify time of day for use. - Identify strategies to overcome self-identified barriers.	
Session 2		
Structure	Content	Printed Material Content
Review	- Reinforce learning from session 1 (ways to relax).	*Tip Sheet 2.1: Steps to Dialogue* - Actively listen to identify thoughts and feelings - Acknowledge the feeling - Name the feeling - Acknowledge your AYAs’ wishes (even if they are impossible) *Session 2 Take Home Messages* - Managing emotions - Understanding double protection - Using steps to dialogue
Managing emotions in the presence of AYA	- Explore ways parents manage overwhelming emotions. - Describe strategies to manage emotions in the moment. - Coach parent enactment of brief emotion management strategies (eg, relaxation or self-distraction strategies, briefly stepping away).	
Double protection: AYA and parent protection from distress	- Query parent about their experiences of watchfulness and vigilance over their AYA. - Discuss common AYA responses. - Acknowledge emotional costs (ie, AYA withdrawing, parent helplessness).	
Steps to dialogue: how to listen so AYA will talk	- Describe and discuss the steps to dialogue: - Actively listen. - Acknowledge feelings. - Give the feelings a name. - Acknowledge your AYA’s wishes (even if it is something impossible). - Role-play steps to dialogue.	
Stumbling blocks to open dialogue	- Discuss potential stumbling blocks to open dialogue. - Reflect on possible differences in [AYA] responses to each role.	
Wrap-up	Using *Session 2.1 Tip Sheet*, develop a prescription for parent to use and practice steps to dialogue to initiate a conversation with [AYA].	
Session 3		
Structure	Content	Printed Material Content
Introduction	- Review sessions 1 and 2 content. - Determine whether or not parent watched AYAs’ therapeutic music video (TMV) project. - Review *Session 3 Worksheet: Paths to Well-being*.	*Session 3 Worksheet: Paths to Well-being* Two versions: - (version 1) Parent watched AYAs’ video. - (version 2) Parent has not watched AYAs’ video. *Session 3 Parent Worksheet: Celebration of Accomplishments* - Brief summary of each session - Reflection on what parent learned and accomplished
Paths to well-being (parent watched TMV)	- Parent perspectives of AYA video. - Overview of RIM resilience risk and protective factors. - Use *Session 3 Worksheet: Paths to Well-being* as a guide to discuss and document messages parents identified in their AYAs’ video based on RIM factors. Examples of AYA song lyrics indicating RIM factors: Risk Factor (Illness-Related Distress): despair in lyrics (eg, “not one more time) Protective Factor (Family Support): (eg, “I've got all the help one person could need”) Resilience Outcome: (eg, “…what would make me feeling this way? My courage, my heart, my God - talkin’ bout my fight…”) -Positive feedback on insights gained.	
Paths to well-being (parent did not watch TMV)	- Discussion of AYA decision not to share video - Use *Session 3 Worksheet Paths to Well-being* as a guide for parent to identify and write observations of RIM strengths they have observed in AYAs and/or their concerns. - Positive feedback on insights.	
Putting it all together through role play	- Review *Tip Sheet 2.1: Steps to Dialogue* worksheet. - Parent develops 2 open-ended questions with intervener support. - Role-play both scenarios. - Help parents reflect (eg, how do you think that went?). - Give positive feedback. - Celebrate accomplishments.	
Wrap-up	- Review *Session 3 Take Home Messages*. - Express gratitude for parent participation.	

Abbreviations: AYA, adolescent/young adult; TMV, therapeutic music video (intervention).

#### PARENT ATTENTION CONTROL CONDITION

Parents assigned to attention control received 2 sessions with a trained interventionist. Session 1 was delivered in person, within 2 weeks of T1 measures. During session 1, parents received a list of several websites that offer information aimed at helping parents support AYAs through the cancer experience. During the session, the interventionist provided the website list, assessed parent access to and comfort with using the Internet, offered supportive instruction as needed, reviewed listed websites, and helped parents develop a plan to explore sites before session 2. Session 2 was delivered by telephone, approximately 7 to 10 days after session 1 (corresponding with AYA session 2). During session 2, the interventionist inquired about websites the parent visited, answered any questions about information found on the websites, and asked about the value and relevance of the information to their own experience. If the parent had not visited any websites, the intervener asked the parent what reasons or barriers kept them from accessing the websites and offered assistance to help parents overcome barriers.

#### AYA TMV INTERVENTION

All AYAs received 5 tailored 60-minute sessions within a 6- to 8-week period with a board-certified music therapist. Intervention structure and tailored delivery were based on Robb’s^28,39,40^ Contextual Support Model. The TMV included therapist-guided experiences with songwriting and video production to help AYAs explore, identify, and express what is important to them during treatment for a life-threatening illness. Sessions 1 to 3 included singing, brainstorming, lyric writing, discussion, and song recording. Session 4 used AYA-developed song lyrics as a foundation for selecting visual video content (eg, artwork, photographs) through storyboarding and discussion. In session 5, participants viewed their video and had the option of sharing their project with family, friends, and/or hospital staff through a “video premier.” Parent presence and involvement (if any) during all sessions were directed by the AYA (Table 2).

**Table 2:** TMV Intervention: Summary of Contextual Support and Intervention Content

Elements of Contextual Support From CSM-MT	Summary of Intervention Content by Session^a^	
	Session	TMV Intervention Content
Structure • Familiar, predictable music • Song Scripts • Storyboards • Leveled involvement Autonomy support • AYA directed • Choices (music, lyrics, visual images, vocalists, involving others) • Quality product Relationship support • Music to communicate unspoken thoughts, feelings, and dreams for the future • AYA centered • Therapist support • Family, peer, and healthcare provider involvement (as determined by AYA)	1	- View prototype video and discuss goals for the session - Learn how to use a songwriting script - Select music for project (ie, offered 10 songs from 5 music genres)^b^ - Brainstorm ideas for lyric/video content (ie, what is important to AYA) - Begin writing lyrics to a familiar song using a songwriting script
	2	- Review accomplishments and discuss goals for the session - Write lyrics to a familiar song using a songwriting script - Discuss lyrics and what is important to AYA - Sing/practice song with music accompaniment soundtrack - Select who will sing on the song recording
	3	- Review accomplishments and discuss goals for the session - Sing/rehearse completed song - Discuss AYA thoughts about/reflections on video project - Digitally record vocal soundtrack for video - Listen to AYA vocals mixed with music accompaniment soundtrack^b^ - Introduce storyboarding process (ie, select visual images to go with song lyrics) - Digital camera available to capture images
	4	- Review accomplishments and discuss goals for the session - Listen to/sing completed song and discuss visual images including memories and their importance - Complete storyboard process - Discuss “video premiere” option (ie, option to invite family, friends, and healthcare providers to view completed video project)
	5	- Review accomplishments and discuss goals for the session - Private viewing of music video project - AYA/therapist visit about the final product - Optional “video premiere” - AYAs given copy of their video project

Abbreviations: AYA, adolescent and young adult; CSM-MT, Contextual Support Model of Music Therapy; TMV, therapeutic music video (intervention).

^a^Sessions facilitated by board-certified music therapists.

^b^Digital accompaniment soundtrack purchased for each music video project. Music selections are available upon request.

### Measures

Parent outcomes were measured across 4 domains (distress, family environment, confidence, and quality of life). Table 3 summarizes each measure’s distributional and psychometric properties, including information about the number of items, score range, and how to interpret scores (eg, meaning of high vs low scores). To evaluate parent distress outcomes (ie, anxiety, mood, perceive stress), we used 3 well-validated measures including the Spielberger State-Trait Anxiety Inventory–State Component,^41^ Profile of Mood States-Short Form,^42^ and Perceived Stress Scale.^43^ Parent outcome measures for family environment included the Family Adaptability/Cohesion Scale II,^44^ Parent-Adolescent Communication Scale,^44^ Family Strengths Scale,^44^ and Perceived Social Support–Health Care Providers.^45^ These measures correspond to the RIM latent variable “family environment”^26^ and are the same measures completed by AYAs (see below). To measure parents’ confidence in their ability to parent their AYAs through the cancer experience, the first author developed the Parenting Confidence-Caring for Adolescents/Young Adults during Cancer. For this scale, parents caring for their AYAs report their level of agreement with 11 items using a 5-point Likert scale ranging from “never” to “always.” Scores range from 11 to 55, with higher scores indicating higher confidence. Finally, parent quality of life was measured using the Index of Well-Being.^46,47^

**Table 3:** Psychometric Properties of Measures at Baseline

Measures	No. Items	Possible Range	Mean (SD)	High Score Indicates Greater (or More)…	Cronbach’s *α*
Parent scale measures					
Parent distress					
Spielberger State-Trait Anxiety Inventory–State Component	20	1-4	2.7 (0.6)	Anxiety	0.94
Profile of Mood States-Short Form	37	0-4	2.3 (0.7)	Mood disturbance	0.96
Perceived Stress Scale	14	0-4	2.1 (0.6)	Perceived stress	0.86
Parent family environment					
Family Adaptability/Cohesion Scale II (FACES II) total score	30	1-5	3.8 (0.4)	Cohesion and adaptability	0.88
FACES II–Family Cohesion	16	1-5	4.0 (0.5)	Cohesion	0.84
FACES II–Family Adaptability	14	1-5	3.7 (0.4)	Adaptability	0.78
Parent/Adolescent Communication Total–Parent	20	1-5	3.9 (0.5)	Communication	0.84
Parent/Adolescent Communication Problems–Parent	10	1-5	3.7 (0.7)	Communication	0.78
Parent/Adolescent Communication Openness–Parent	10	1-5	4.1 (0.5)	Communication	0.76
Family Strengths Scale	12	1-5	4.0 (0.6)	Strength	0.79
Perceived Support-Health Care Professional	20	1-5	4.1 (0.6)	Support	0.89
Parent confidence					
Parenting Confidence-Caring for Adolescents/Young Adults during Cancer	11	1-5	4.0 (0.6)	Confidence	0.90
Parent quality of life					
Index of Well-Being	9	1-7	5.5 (1.0)	Well-being	0.88
AYA latent variable measures					
Latent variable: AYA family environment					
Family Adaptability/Cohesion Scale II (FACES II) total score	30	1-5	3.6 (0.5)	Cohesion and adaptability	0.90
FACES II–Family Cohesion	16	1-5	3.7 (0.6)	Cohesion	0.84
FACES II–Family Adaptability	14	1-5	3.4 (0.6)	Adaptability	0.84
Parent/Adolescent Communication–Total	20	1-5	3.7 (0.6)	Communication	0.88
Parent/Adolescent Communication–Problems	10	1-5	3.3 (0.7)	Communication	0.79
Parent/Adolescent Communication–Openness	10	1-5	4.1 (0.6)	Communication	0.90
Family Strengths Scale	12	1-5	3.8 (0.6)	Family strengths	0.84
Latent variable: courageous (positive) coping					
Jalowiec Coping Scale Part A (revised): Confrontive, Optimistic, and Supportant Coping subscales	10	0-3	2.2 (0.5)	Courageous coping	0.73
Latent variable: hope-derived meaning					
Hearth Hope Index					
Expectancy/Interconnectedness subscale	4	0-3		Hope	0.50
Positive Readiness subscale	4	0-3			0.55
Latent variable: resilience resolution					
Haase Resilience in Illness Scale	15	1-6		Resilience	0.76

Abbreviation: AYA, adolescent and young adult.

Adolescent and young adult outcome measures for “family environment,” “positive coping,” “hope-derived meaning,” and “resilience resolution” correspond to RIM latent variables, validated with confirmatory structural equation modeling.^25^ Table 3 summarizes each measure’s distributional and psychometric properties. Cronbach’s *α* coefficients from T1 data ranged from .79 to .96 (.76-.84 for subscales). More detailed descriptions of the “family environment,” “positive coping,” “hope-derived meaning,” “resilience resolution,” and quality of life measures are available in exploratory and confirmatory RIM evaluation reports.^25,45^

### Statistical Analyses

SAS software version 9.4 was used for all analyses.^48^ Between-group comparisons on parent outcomes for the intervention and attention control groups were performed using analysis of covariance (ANCOVA). Randomized groups were compared separately at T2 and T3, controlling for T1 variables. Before running ANCOVA models, we compared groups on baseline variables to determine which variables to include as covariates in models. If the comparison test exhibited a *P* value less than .20, the covariate was included in the model to control for potential confounding. Adjusted means from the ANCOVA models were then used with Cohen’s *d* (standardized mean difference between groups) to calculate an effect size (ES) for the between-group difference. Cohen’s *d* was defined as the mean difference between groups divided by the pooled standard deviation across groups. Our study was powered for 64 parents in each group at baseline and T2 to detect a medium ES of 0.50. Within-group comparisons were also performed by calculating the standardized response mean (SRM) ES and paired *t* test for the intervention and attention control groups separately. The formula for SRM was defined as the mean difference between T1 and T2 or T1 and T3 (depending on the comparison) divided by the standard deviation of the difference.

Because the latent variable RIM model is well validated for AYAs, outcomes for AYAs were tested using a latent-variable ANCOVA approach. This approach was also used to account for measurement error and to reduce the number of comparisons. Mplus software version 8.1 was used for all models, specifying maximum likelihood estimation. We compared the randomized groups on each latent variable at T2 adjusted for the corresponding latent variable at T1, and on each latent variable at T3 adjusted for the same latent variables at T1, using scale scores as observed indicators of the latent variables as specified in the RIM measurement model. To provide meaningful interpretations, the ES for the intervention effect on the T2 and T3 latent variables was computed by converting the *t* value of the intervention coefficients supplied by Mplus output to a Cohen standardized difference between means.^49^

### Qualitative Analysis

Digital recordings of interviews were transcribed verbatim, deidentified, and checked for accuracy. Qualitative data were analyzed by the second and third authors using deductive content analysis.^50^ With deductive content analysis, the focus is on how data inform the research questions rather than inferring new relationships. The interview guide explored these concepts from the participants’ perspective, and data analysis focused on how participant perceptions informed the research questions.

## Results

Table 2 summarizes study accrual, intervention delivery, and data collection. Parent and AYA demographics are summarized in Table 4. Parents’ sex and race, AYAs sex, and whether AYAs were attending school were potentially confounding baseline covariates; they differed at least marginally between groups at baseline (*P* < .10; Table 4).

**Table 4:** Baseline Characteristics by Study Group

	TMV + Parent Attention Control		TMV + Parent Intervention		Overall		*P* ^a^
	*n*	Mean/%	*n*	Mean/%	*n*	Mean/%	
Parent demographics							
Age, mean (SD), range, y	54	43.4 (7.2), 28-57	56	42.9 (7.7), 30-62	110	43.2 (7.4), 28-62	.7269
Sex, %	54		56		110		.0433
Female		79.6		92.9		86.4	
Male		20.4		7.1		13.6	
Race, %	54		55		109		.0620
African American		7.4		16.4		11.9	
White		77.8		80.0		78.9	
Other^b^		14.8		3.6		9.2	
Ethnicity, %	53		56		109		.3208
Hispanic		15.1		8.9		11.9	
Not Hispanic		84.9		91.1		88.1	
Years of education, mean (SD), range	54	14.5 (1.9), 12-17	56	14.3 (1.8), 10-17	110	14.4 (1.9), 10-17	.6539
Employment status, %	54		55		109		.1506
Employed (full- or part-time)		50.0		63.6		56.9	
Not employed		50.0		36.4		43.1	
Household income, %	53		55		108		.3735
<$25 000		30.2		27.3		28.7	
$25 000-$50 000		26.4		14.5		20.4	
$50 000-$100 000		26.4		36.4		31.5	
>$100 000		17.0		21.8		19.4	
AYA Demographics							
Age, mean (SD), range, y	54	15.0 (2.9), 11-22	56	15.4 (3.4), 11-24	110	15.2 (3.2), 11-24	.5664
Sex, %	54		56		110		.0880
Female		46.3		62.5		54.5	
Male		53.7		37.5		45.5	
Race, %	54		55		109		.2392
African American		7.4		12.7		10.1	
White		59.3		67.3		63.3	
Other^b^		33.3		20.0		26.6	
Ethnicity, %	54		56		110		.3391
Hispanic		14.8		8.9		11.8	
Not Hispanic		85.2		91.1		88.2	
Years of education, mean (SD), range	54	8.3 (2.6), 4-14	56	8.7 (2.9), 5-16	110	8.5 (2.8), 4-16	.4921
Currently attending school, %	54		56		110		.0911
No		16.7		30.4		23.6	
Yes		83.3		69.6		76.4	
Employment status, %	54		55		109		.6775
Employed (full- or part-time)		7.4		5.4		6.4	
Not employed		92.6		94.5		93.6	

Abbreviations: AYA, adolescent and young adult; TMV, therapeutic music video.

Parent demographics are reported on the primary parent of AYA.

^a^Study group comparisons were made using χ^2^ (all categorical) and *t* (age and education) tests.

^b^Includes American Indian, Alaskan Native, Asian, Native Hawaiian, Pacific Islander, and other races.

### Parent Outcomes

The intervention and attention control groups did not exhibit significant between-group differences on primary parent outcomes of distress, family environment, and quality of life (ie, sense of well-being) at T2 (14 days post intervention) or T3 (90 days post intervention), while adjusting for the baseline measure of the outcome and the potentially confounding baseline covariates mentioned previously (Table 5). However, for the secondary outcome Parenting Confidence-Caring for Adolescents/Young Adults during Cancer, there was a significant between-group difference that favored the intervention group (Table 5). Specifically, compared with the control group, the intervention group demonstrated significantly (*P* = .04) better outcomes for parenting confidence at T2 with a moderate ES (Cohen’s *d* was 0.53 in absolute value). In addition, at T3, the intervention group demonstrated marginally (*P* < .10) better outcomes for family adaptability/cohesion (FACES II total score) with a moderate ES (Cohen’s *d* was 0.44 in absolute value).

**Table 5:** Within-Group and Between-Group Comparisons for Parent Outcomes

Variable	Within-Group Effects^a^								Between-Group Effects^b^		
	TMV + Parent Attention Control (n = 37)				TMV + Parent Intervention (n = 30)						
	Mean (SD)	SRM^c^	95% CI	*P* ^d^	Mean (SD)	SRM^c^	95% CI	*P* ^d^	*d* ^e^	95% CI	*P* ^f^
Spielberger State Anxiety (higher = worse)											
Baseline	2.25 (0.68)				2.33 (0.53)						
14 d post intervention	2.11 (0.69)	−0.23	(−0.56 to 0.10)	.1686	1.91 (0.61)	−0.60	(−0.97 to −0.23)	.0027	−0.39	(−0.90 to 0.11)	.1295
90 d post intervention	1.94 (0.69)	−0.53	(−0.87 to −0.20)	.0025	1.88 (0.65)	−0.59	(−0.96 to −0.21)	.0032	−0.19	(−0.69 to 0.31)	.4588
Perceived stress (higher = worse)											
Baseline	1.98 (0.59)				1.99 (0.48)						
14 d post intervention	1.72 (0.63)	−0.49	(−0.82 to −0.15)	.0055	1.66 (0.71)	−0.47	(−0.84 to −0.10)	.0156	−0.12	(−0.63 to 0.38)	.6277
90 d post intervention	1.64 (0.65)	−0.56	(−0.89,- 0.23)	.0016	1.57 (0.63)	−0.54	(−0.91 to −0.16)	.0064	−0.19	(−0.70 to 0.31)	.4514
Mood state, total score (higher = worse)											
Baseline	1.76 (0.74)				1.78 (0.61)						
14 d post intervention	1.49 (0.74)	−0.49	(−0.82 to −0.15)	.0055	1.32 (0.73)	−0.63	(−1.00 to −0.26)	.0017	−0.32	(−0.83 to 0.18)	.2100
90 d post intervention	1.37 (0.75)	−0.60	(−0.93 to −0.27)	.0008	1.18 (0.72)	−0.71	(−1.08 to −0.34)	.0006	−0.36	(−0.86 to 0.15)	.1691
FACES II–Family Cohesion (higher = better)											
Baseline	3.90 (0.47)				3.94 (0.49)						
14 d post intervention	3.93 (0.49)	0.11	(−0.23 to 0.44)	.5230	3.94 (0.45)	0.01	(−0.37 to 0.38)	.9718	−0.08	(−0.58 to 0.42)	.7581
90 d post intervention	3.94 (0.47)	0.14	(−0.20 to 0.47)	.4059	4.08 (0.31)	0.31	(−0.06 to 0.68)	.0994	0.40	(−0.11 to 0.90)	.1268
FACES II–Family Adaptability (higher = better)											
Baseline	3.60 (0.35)				3.61 (0.48)						
14 d post intervention	3.58 (0.41)	−0.05	(−0.39 to 0.28)	.7545	3.64 (0.38)	0.09	(−0.28 to 0.47)	.6181	0.12	(−0.38 to 0.62)	.6411
90 d post intervention	3.61 (0.35)	0.04	(−0.29 to 0.38)	.7994	3.70 (0.37)	0.20	(−0.17 to 0.58)	.2744	0.40	(−0.10 to 0.90)	.1209
FACES II mean total score (higher = better)											
Baseline	3.76 (0.35)				3.78 (0.45)						
14 d post intervention	3.77 (0.43)	0.03	(−0.30 to 0.37)	.8433	3.80 (0.39)	0.05	(−0.32 to 0.43)	.7745	0.02	(−0.48 to 0.52)	.9465
90 d post intervention	3.79 (0.38)	0.11	(−0.23 to 0.44)	.5180	3.90 (0.39)	0.28	(−0.09 to 0.65)	.1367	0.44	(−0.06 to 0.94)	.0915
Parent Adolescent Communication Total-Parent (higher = better)											
Baseline	3.83 (0.50)				3.81 (0.54)						
14 d post intervention	3.82 (0.56)	−0.03	(−0.36 to 0.31)	.8710	3.76 (0.52)	−0.12	(−0.49 to 0.25)	.5169	−0.03	(−0.53 to 0.47)	.9035
90 d post intervention	3.83 (0.53)	0.02	(−0.31 to 0.35)	.9088	3.80 (0.63)	−0.02	(−0.40 to 0.35)	.8959	0.04	(−0.47 to 0.54)	.8882
Parent Adolescent Communication Problems-Parent (higher = better)											
Baseline	3.65 (0.58)				3.58 (0.77)						
14 d post intervention	3.59 (0.63)	−0.12	(−0.45 to 0.21)	.4649	3.55 (0.78)	−0.08	(−0.45 to 0.29)	.6645	0.06	(−0.44 to 0.57)	.8074
90 d post intervention	3.60 (0.65)	−0.08	(−0.42 to 0.25)	.6193	3.60 (0.75)	0.04	(−0.33 to 0.41)	.8341	0.07	(−0.44 to 0.57)	.7986
Parent Adolescent Communication Openness-Parent (higher = better)											
Baseline	4.01 (0.56)				4.03 (0.46)						
14 d post intervention	4.05 (0.60)	0.13	(−0.20 to 0.47)	.4227	3.98 (0.49)	−0.11	(−0.48 to 0.27)	.5609	−0.14	(−0.64 to 0.36)	.5823
90 d post intervention	4.06 (0.55)	0.17	(−0.16 to 0.51)	.2970	4.00 (0.63)	−0.06	(−0.44 to 0.31)	.7314	−0.03	(−0.53 to 0.47)	.9005
Family strengths (higher = better)											
Baseline	3.82 (0.64)				3.89 (0.55)						
14 d post intervention	3.93 (0.68)	0.23	(−0.11 to 0.56)	.1760	3.91 (0.47)	0.06	(−0.31 to 0.43)	.7404	−0.18	(−0.68 to 0.32)	.4903
90 d post intervention	3.98 (0.61)	0.34	(0.01 to 0.67)	.0463	3.96 (0.52)	0.18	(−0.19 to 0.56)	.3206	−0.15	(−0.66 to 0.35)	.5511
Perceived Support-Health Care Professional (higher = better)											
Baseline	4.10 (0.55)				4.06 (0.61)						
14 d post intervention	4.09 (0.72)	−0.01	(−0.35 to 0.32)	.9438	4.05 (0.68)	−0.01	(−0.38 to 0.37)	.9736	0.05	(−0.45 to 0.55)	.8474
90 d post intervention	4.07 (0.70)	−0.06	(−0.40 to 0.27)	.7022	4.01 (0.70)	−0.10	(−0.47 to 0.27)	.5849	0.02	(−0.48 to 0.52)	.9403
Confidence (higher = better)											
Baseline	3.99 (0.54)				4.00 (0.51)						
14 d post intervention	3.96 (0.55)	−0.09	(−0.43 to 0.24)	.5682	4.15 (0.45)	0.32	(−0.05 to 0.70)	.0887	0.53	(0.03-1.03)	.0430
90 d post intervention	4.03 (0.49)	0.10	(−0.24 to 0.43)	.5561	4.07 (0.51)	0.16	(−0.22 to 0.53)	.3948	0.08	(−0.42 to 0.58)	.7483
Index of Well Being (higher = better)											
Baseline	5.50 (1.02)				5.50 (0.96)						
14 d post intervention	5.61 (1.12)	0.13	(−0.21 to 0.46)	.4498	5.54 (1.03)	0.06	(−0.32 to 0.43)	.7603	−0.03	(−0.53 to 0.47)	.9166
90 d post intervention	5.70 (0.96)	0.28	(−0.05 to 0.62)	.0947	5.63 (1.28)	0.11	(−0.27 to 0.48)	.5640	−0.01	(−0.52 to 0.49)	.9540

Abbreviations: CI, confidence interval; FACES, Family Adaptability/Cohesion Scale; SRM, standardized response mean; TMV, therapeutic music video.

^a^Raw means and standard deviations are included in the table.

^b^Values were calculated from a linear regression model of follow-up scores adjusting for baseline covariates (adolescent and young adult [AYA] sex, parent sex, parent race, and whether AYA was attending school) and baseline version of the outcome (ANCOVA).

^c^Standardized response mean calculated as mean change score (follow-up minus baseline) divided by the standard deviation of the change score; positive sign for SRM indicates a higher follow-up score compared with the baseline score.

^d^*P* value calculated from a 2-sided paired *t* test within the attention control and treatment groups.

^e^Cohen *d* effect size calculated as the difference in adjusted means between the intervention groups at follow-up divided by the pooled standard deviation of scores at follow-up. A positive (negative) Cohen *d* value indicates the time 2 or time 3 adjusted mean was higher (lower) for TMV + parent group compared with the TMV + low-dose control group.

^f^*P* value from ANCOVA for the 2-sided test of the effect of treatment versus attention control at follow-up adjusted for baseline covariates.

Both parent groups exhibited significant within-group improvement, especially for measures of parent distress including anxiety, perceived stress, and mood (Table 5). Specifically, both groups showed statistically significant within-group improvement (*P* < .05) in state anxiety (T3), perceived stress (T2 and T3), and mood (T2 and T3) with moderate SRM ESs ranging between 0.47 and 0.71 in absolute value. In addition, state anxiety improved at T2 for the intervention group (SRM = −0.60, *P* = .0027), and the improvement was not statistically significant for the attention control group (SRM = −0.23, *P* = .1686). Whereas improvement at T3 for family strengths was statistically significant for the attention control group (*P* = .0463), it was not significant for the intervention group (*P* = .3206).

### AYA Outcomes

Adolescent and young adult groups did not differ significantly for any of the outcomes (*P* > .60 for T2 and *P* > .13 for T3), and Cohen’s *d* ESs were small (Table 6).

**Table 6:** Effect Size Measures for Latent Variable AYA Outcomes Obtained From Structural Equation Models

Factor	Time 2					Time 3				
	n_1_	n_2_	*t*	Cohen’s Effect Size	*P*	n_1_	n_2_	*t*	Cohen’s Effect Size	*P*
Family environment	30	41	−0.522	−0.127	.602	32	40	−1.161	−0.279	.246
Positive coping	31	41	−0.185	−0.045	.853	32	40	−0.835	−0.201	.404
Hope-derived meaning	31	41	−0.261	−0.063	.794	32	40	−1.511	−0.363	.131
Resilience	31	40	−0.018	−0.004	.986	32	40	0.11	0.026	.912

Abbreviation: AYA, adolescent and young adult.

n_1_ indicates the sample size for the treatment group; n_2_ indicates the sample size for the low-dose control group. *t* indicates *t* value for the treatment effect obtained from Mplus output and defined as the ratio of unstandardized estimate/standard error. Cohen effect size is defined as follows: t*[SQRT([(n_1_ + n_2_)/(n_1_*n_2_)]*[(n_1_ + n_2_)/(n_1_ + n_2_ − 2)])] (see Thalheimer and Cook [2002]). A positive (negative) sign for the *t* value and effect size implies that the time 2 or time 3 adjusted mean for the latent factor was higher (lower) for the treatment group compared with the low-dose control group. Models were adjusted for the following covariates: parent race, parent sex, AYA sex, and whether AYA was attending school.

### Parent Interviews

Overall, parents benefited from the intervention and found it helpful (see Table 7 for categories, subcategories, and exemplar quotes). However, parents noted that there were limits to what a relatively short intervention could provide and that some content was inconsistent with their ways of coping and communication (eg, talking can be “emotionally taxing”) or touched on skills they felt were already strong (eg, “had a pretty good relationship going in”). The most beneficial aspect of the parent intervention was the opportunity to receive support for themselves. Several parents mentioned that scheduled time with the nurse intervener was a study expectation and, therefore, provided a “pass” to take some time for themselves. Some felt guilty about leaving their AYAs when they were not feeling well but appreciated the opportunity to have someone who understands what is going on spend some time supporting them.

**Table 7:** Qualitative Data Characterizing Parent Benefit

Categories	Subcategories	Exemplar Quotes
Parent distress	Timing of support	“We were in 18 months already through chemo and all that and probably the study more upfront would have been helpful, but I realize that’s when it came out.” (Site5Parent3)
	Parenting support	“I think it helped me have someone to talk to in the hospital, bounce ideas when I was lost for what to do and where to turn. It made me feel like I wasn’t alone in the experience.” (Site2Parent8)
	Relaxation strategies	“Take 10 minutes and just breathe because that’s the first thing you forget, especially when things are tense.” (Site1Parent2)
	Emotional needs (met)	“There are a lot of emotional things that a parent goes through when a child is diagnosed with cancer, and you really do not know what type of support is available to you. You do not know how to handle a lot of circumstances, issues that come up, and just going through the questions and learning ways to handle and adapt to various situations was very helpful.” (Site1Parent29)
	Emotional needs (unmet)	“For me the worst thing was doing the meetings because it was so emotionally draining. I mean overall I think it’s great because there’s a lot of advice…. Doing that was great and there were a lot of things that I did learn and different ways to say things at different times to listen and things like that. Maybe because I do not talk about it because I cannot. It was emotionally taxing. I was exhausted afterwards.” (Site2Parent14)
Parent-AYA relationship	Increased connection	“I think there is more of a connection. I think I understand her a little bit better.” (Site1Parent7)
	Already strong relationship	“I do not know that it changed it any. I felt like we had a pretty good relationship going in. Like I said, it did give me some ideas about how to approach some things with him. So in that way it probably helped some but I do not think that the relationship changed any.” (Site1Parent31)
Parent-AYA communication		“I think because of asking those types of questions, she’s been more forthcoming with information without having to ask, because she feels that there is, I do care, and that there is a safety net there…. I thought oh, my gosh, she really trusts and values my opinion.” (Site1Parent29)
Sense of well-being		“I think it was just kind of nice to have someone talking with me and just focus on me instead of everything on my child. I appreciated that.” (Site4Parent5)
		“It was like an excused absence and you could go and just really, really get a fresh perspective, really have a chance to say whatever you want, have an opportunity to cry and get pulled back together again before you went back in the room.” (Site1Parent2)
Perceived social support from healthcare providers		“You felt like oh my Gosh, I cannot believe that, having to go through this, but you guys made me feel like, I do not know, just like I wasn’t alone.” (Site3Parent6)
		“I think just having somebody there. At least I would do some of that and all the techniques were helpful. I cannot name them all but I just feel like just having somebody with you or to kind of listen to me or I mean it was just wonderful to have that available.” (Site1Parent41)
Parent experience relief from AYA involvement in TMV	Expanding AYA social support network	“(TMV was great because) as a parent you do feel drained after awhile if you are their only support. You need help in that area because you cannot possibly be everything that your child needs for support.” (Site1Parent2)
	Relief that AYA had something positive in the midst of cancer	“Just knowing that she had something to put focus on, put her mind on, and give her something to look forward to…. She would get excited and she would smile, and she would talk about the photos that she wanted to put in it.” (Site3Parent6)
	TMV as a window into AYA’s experience	“I thought it was interesting to see what AYA thought, his thought process, and had problems dealing with things.” (Site5Parent2)

Abbreviations: AYA, adolescent and young adult; TMV, therapeutic music video.

For self-care content, parents appreciated opportunities to learn relaxation and coping skills to manage their own distress. For parent-AYA communication content, most parents perceived that their relationship and communication with their AYA was fairly good already but appreciated learning skills to further enhance their communication. In relation to the TMV, parents experienced relief in knowing that their AYAs were receiving support from the music therapist. For some, this lifted the weight of being their child’s primary source of support. Relief also came from recognizing that their child had an outlet for self-expression during this challenging time. Several parents suggested that they would have appreciated having the intervention sooner in the treatment trajectory, for both themselves and their AYAs, but acknowledged that they might not have been receptive to the intervention earlier.

## Discussion

Open parent-AYA communication about cancer-related concerns and the future is important for both short- and long-term outcomes for parents and AYAs. However, these conversations can be difficult due to high parent and AYA distress and are often avoided in an attempt to protect one another from further distress.^12,14,21–24^ In this study, which was based on the RIM for AYAs with cancer and findings from our previous trials,^25,28,29^ we evaluated a “self-care and communication” intervention for parents to help them manage their own distress and engage in open dialogue about what was important to their AYAs.

For parents, we hypothesized that the intervention would significantly decrease primary outcomes of parent distress, enhance family environment, and improve well-being compared with the control condition. However, we found no significant between-group differences on our primary outcomes of interest, with both groups exhibiting significant within-group improvement on all measures of parent distress. We did find significant differences that favored the parent intervention group on parenting confidence at T2 and marginally better outcomes for family adaptability/cohesion at T3. For AYAs, we hypothesized that, by lowering parent distress and improving parent communication skills, AYAs would experience an additional benefit beyond receipt of TMV alone. Given the absence of between-group differences for parents, the lack of between-group differences for AYAs was not surprising.

For parents, our qualitative interview data support our quantitative findings that, as a brief intervention, the “self-care and communication” intervention was successful in raising self-awareness and parent confidence in the short term. Parents indicated that the strategies they learned were helpful and that they derived benefit from relaxation and communication session content. However, the most impactful part of the intervention was having dedicated time to talk about their own needs with nurse interveners who understood the cancer experience. In addition, for some parents, certain content was not consistent with their immediate needs, ways of coping, and/or communicating—suggesting the need for an adaptive intervention.^51,52^ Of particular importance to clinical care, parents identified their own need for communication and support from nurses. The focus of care is often on the AYA, but to optimize the care of the AYA, the caregiver needs attention as well. Parents would benefit from ongoing communication and encouragement to care for themselves in addition to their AYAs from nurses.

Three potential reasons for the lack of between-group differences include the need for a more sustained intervention period with tailored delivery,^52,53^ insufficient power, and an indirect benefit that parents in the control group may have derived from the TMV. First, the parent intervention was designed to address 2 areas of parent need—self-care and communication. The parent intervention was composed of three 1-hour sessions (one focused on self-care and two focused on fostering parent-AYA communication). Although our interview data indicate parents found benefit, acquisition of relaxation and communication skills requires time and practice. Parents shared that there was not enough time to develop and apply the skills they were learning, making it easy to slip into old habits. It is likely that 3 sessions did not provide enough time to teach and support parents’ efforts to practice and apply these skills. Some parents shared that they already had strategies to support their relationship and communication with their AYAs, such that they viewed much of the content as “a good reminder” about what they could do, whereas others shared that, although session content was beneficial, talking about their AYAs’ experience could be emotionally taxing. These findings suggest the need for an adaptive intervention where parents can select topics, skills, and content based on their self-identified needs. Use of sequential multiple randomization trial (SMART) designs to develop and optimize adaptive interventions gained visibility during the conduct of this trial.^54^ Use of a SMART design for subsequent trials would support identification of decision rules that link characteristics of the individual parent (eg, preferences, communication style, and ways of coping) with specific intervention components delivered at the optimal frequency.^51–54^

The second potential reason for lack of significant between-group differences is insufficient power. Our study was powered for 64 parents in each group at baseline (T1) and T2 to detect a medium ES (*d* = 0.50). However, recruiting AYAs and parents as a dyad required mutual consent that may have contributed to the final sample size being 52% of planned enrollment. Despite being underpowered, the significant between-group difference favoring the parent intervention on the parenting confidence measure (2 weeks post intervention) and marginal between-group differences on “family adaptability and cohesion” (90 days post intervention), coupled with parent interview data, suggests that parents derived some benefit from the intervention. However, we acknowledge that the validity and reliability of the Parenting Confidence-Caring for Adolescents/Young Adults during Cancer are not fully established, so these findings must be interpreted with caution. In addition, challenges with dyad recruitment may suggest greater potential for selection bias. At the time of study enrollment, parents and AYAs with good existing communication skills or more time may have been more likely to participate.

The third reason for diminished between-group differences is that parents in both groups may have derived an indirect benefit from their AYAs’ involvement with the TMV intervention. In our previous trial, parents enjoyed viewing and/or becoming involved in their AYAs’ TMV video project because it provided an opportunity to better understand how their AYAs were coping with their cancer experience and opportunities for meaningful connection.^38^ This experience may have created a sense of relief that manifested in lower anxiety, perceived stress, and improved mood for parents. This is consistent with parent interview data for this trial wherein parents noted that the TMV intervention provided support and a voice to their AYAs. The receipt of this support allowed parents to feel less like they were wholly responsible for supporting their AYAs through the treatment process. In addition, the AYAs’ video project gave some parents insight into their AYAs’ thoughts and feelings, providing information to better inform the support parents were providing. This is suggestive of indirect benefit; however, the trial was not designed to isolate and examine indirect benefits of the TMV for parents.

On the basis of these findings, we recommend that future studies of the “self-care and communication” intervention maintain use of scheduled appointments with a nurse interventionist. Parents characterized this as a positive form of “accountability” that helped them prioritize their own needs when this feels counterintuitive. As caregivers of seriously ill AYAs, parents often neglect their own needs and find it hard to accept care focused on themselves.^55,56^ Because content was helpful, but not always consistent with a parent’s most pressing need, we recommend tailored delivery of the intervention. These 2 recommendations are grounded in statements from parents that having a designated person and time away to meet their own needs was a valuable benefit of the study. The content was appreciated, but the time and support were even more valuable. Many parents noted that having “someone who understood what they were going through” to whom they did not have to explain and did not feel judged by helped mitigate their distress. We also recommend a longer intervention period to provide sustained support in the form of active listening and greater acquisition of relaxation and communication skills, which requires time and practice. Finally, to provide greater flexibility and standardization of communication content and delivery, we recommend evaluating the parent intervention using a SMART design independent of the TMV.^51–54^

In summary, despite the importance and need for parent-AYA communication interventions, few exist and, those that do, center on treatment or care planning decisions.^57,58^ Parent interview data from this trial increased our understanding about the needs of parents and their perspectives, which can directly inform refinement of the “self-care and communication” intervention. We recommend further research to strengthen the intervention in terms of timing, duration, and tailored content to better meet the needs of parents of AYAs with cancer. A separate trial to examine indirect benefits that parents may be deriving from AYAs’ receipt of the TMV intervention would also be appropriate.
